# Highly selective reduced graphene oxide (rGO) sensor based on a peptide aptamer receptor for detecting explosives

**DOI:** 10.1038/s41598-019-45936-z

**Published:** 2019-07-16

**Authors:** Kyungjae Lee, Yong Kyoung Yoo, Myung-Sic Chae, Kyo Seon Hwang, Junwoo Lee, Hyungsuk Kim, Don Hur, Jeong Hoon Lee

**Affiliations:** 1 Department of Electrical Engineering, Kwangwoon University, 447-1 Wolgye, Nowon, Seoul 01897 South Korea; 2 Department of Clinical Pharmacology and Therapeutics, College of Medicine, Kyung Hee University, Seoul, 02447 South Korea

**Keywords:** Sensors, Electronic properties and devices

## Abstract

An essential requirement for bio/chemical sensors and electronic nose systems is the ability to detect the intended target at room temperature with high selectivity. We report a reduced graphene oxide (rGO)-based gas sensor functionalized with a peptide receptor to detect dinitrotoluene (DNT), which is a byproduct of trinitrotoluene (TNT). We fabricated the multi-arrayed rGO sensor using spin coating and a standard microfabrication technique. Subsequently, the rGO was subjected to photolithography and an etching process, after which we prepared the DNT-specific binding peptide (DNT-bp, sequence: His-Pro-Asn-Phe-Se r-Lys-Tyr-IleLeu-HisGln-Arg-Cys) and DNT non-specific binding peptide (DNT-nbp, sequence: Thr-Ser-Met-Leu-Leu-Met-Ser-Pro-Lys-His-Gln-Ala-Cys). These two peptides were prepared to function as highly specific and highly non-specific (for the control experiment) peptide receptors, respectively. By detecting the differential signals between the DNT-bp and DNT-nbp functionalized rGO sensor, we demonstrated the ability of 2,4-dinitrotoluene (DNT) targets to bind to DNT-specific binding peptide surfaces, showing good sensitivity and selectivity. The advantage of using the differential signal is that it eliminates unwanted electrical noise and/or environmental effects. We achieved sensitivity of 27 ± 2 × 10^−6^ per part per billion (ppb) for the slope of resistance change versus DNT gas concentration of 80, 160, 240, 320, and 480 ppm, respectively. By sequentially flowing DNT vapor (320 ppb), acetone (100 ppm), toluene (1 ppm), and ethanol (100 ppm) onto the rGO sensors, the change in the signal of rGO in the presence of DNT gas is 6400 × 10^−6^ per ppb whereas the signals from the other gases show no changes, representing highly selective performance. Using this platform, we were also able to regenerate the surface by simply purging with N_2_.

## Introduction

Gas sensors developed to convert chemical information and the concentration of a particular gas into electrical signals have gained significant attention as key sensors in the fields of security, healthcare, environmental monitoring, and energy saving. Importantly, the direct electrical transducing ability of a target binding to a receptor-immobilized sensor has the merits of fast response and high sensitivity, especially for smartphone-based sensors. Chemiresistive gas sensors based on metal oxides have been extensively developed for the electrical detection of gases in environmental monitoring; however, metal-oxide-based gas sensors generally require external/internal heaters to enable the adsorption and desorption of target gas molecules. This increases their power consumption, thereby hindering the practical application of these materials as sensors^1–5^.

Applications for the Internet of Things (IoT) require high sensitivity, precise selectivity, rapid response/recovery, and stability for long-term operation. Another important key parameter for IoT applications is that the power consumption of the sensing device should be low^6,7^. Carbon nanomaterials, such as carbon nanotubes (CNTs) and graphene, have been applied in various devices owing to their excellent electrical and mechanical properties^8–10^. Because graphene is known to respond in a highly sensitive manner, it could have the requisite sensing performance. This prompted many researchers to study graphene sensors^3,11–13^. In recent years, the use of hybrid graphene materials (e.g., Pt, ZnO, TiO_2_, and graphene) as well as several processing methods (e.g., thermal and plasma treatment) for graphene have been reported to achieve high sensitivity^11,14–17^. As an alternative, reduced graphene oxide (rGO) offers the ease of surface modification and functionalization such that rGO-based sensors are widely utilized as biosensors^18–21^.

The increasing threat posed by the use of improvised explosive devices (IEDs) in civilian and military populations has resulted in the extensive investigation of explosive-related chemical vapor sensors^22^. Dinitrotoluene (DNT) is the decomposition product of trinitrotoluene (TNT); thus, the former can be used as an explosive target material instead of the latter. Because an effective sensor for explosives, in particular for DNT detection, is essential for monitoring/controlling dangerous/hazardous environments, the development of DNT gas sensors with satisfactory selectivity as well as the required sensitivity and low power consumption is extremely necessary. However, the development of sensors with these properties continues to remain a challenge that needs to be addressed.

Because most commercially developed receptors have limited selectivity, it is usually necessary to use other analytical techniques such as principal component analysis (PCA) for enhanced selectivity. In this regard, the peptide (aptamer) has received considerable attention as a receptor because of its high selectivity^22–25^. The peptide (aptamer) receptor is considered to be a strong candidate for increasing the selectivity as interactions are based on multivalent or cooperative binding that enable highly specific recognition^25^. Moreover, a receptor based on the peptide (aptamer) could be expected to provide rapid response time and allow facile regeneration of the sensor surface.

We recently demonstrated a sensor with improved selectivity by using a peptide (aptamer) receptor based on a piezoelectric cantilever^22,23,26^. We reported that this cantilever, the effectiveness of which mainly relies on complex microfabrication, showed great improvements in selectivity as well as sensitivity. We fabricated the microcantilever, which was composed of six multilayers, i.e., SiN_x_/Ta/Pt/PZT/Pt/SiO_2_, by using several depositions, photolithography, and an etching process. Moreover, we determined that the residual stress that affects the piezoelectrical characteristics as well as the reliability in the MEMS device had to be handled with care. Failure to control the residual stress was found to prevent successful fabrication because of cracking, bending, and unintended electromechanical operation^27^.

Our attempts to address these problems led us to devise strategies to realize a highly specific electrical detection system using the aforementioned multi-arrayed rGO sensor with a DNT-specific binding peptide. The combination of a highly specific peptide receptor with rGO-based electrical detection enabled us to develop a platform for sensors with high selectivity as well as high sensitivity. Additionally, this device platform is able to provide reproducibility and a regenerated surface for utilization in real field applications.

## Experimental

### rGO sensor fabrication

**Figure 1:**
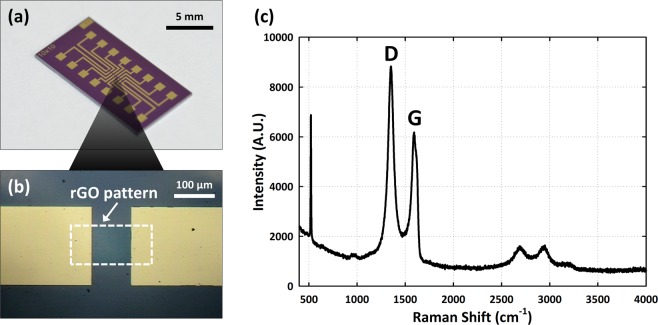
Illustrations showing details of the rGO sensor (**a**) enlargement of the board containing eight arrays for DNT detection; (**b**) location of the rGO sensor (200 μm wide by 100 μm long) between two Au electrodes. (**c**) Raman spectrum of the rGO sensor clearly showing the D and G bands at 1350 cm^−1^ and 1580 cm^−1^, respectively.

We designed the rGO gas sensor with a multi-array as shown in Fig. 1(a). First, a layer of silicon dioxide (SiO_2_) with a thickness of 300 nm was deposited on the silicon substrate by using thermal oxidation, and then graphite powder was used to prepare graphene oxide (GO) flakes with Hummers’ method before rGO deposition. A GO thin film was deposited on the SiO_2_ layer using a spin-coating technique. The GO thin film was then reduced with hydriodic acid (HI) vapor at 80 °C for 3 h. After deposition of the rGO layer, we used standard photolithography to form rGO patterns (MA6 Aligner, Karl Suss) and an etching process (inductively coupled plasma-reactive ion etcher (ICP-RIE, Oxford Inc.)). We deposited the Au electrode (200 nm) with e-beam evaporation and a lift-off process. Figure 1(b) shows the rGO patterns (200 μm × 100 μm) between the Au electrodes. The fabrication process of the rGO sensor is shown in more detail in Fig. S1 of the supplementary information. The Raman spectrum of the rGO sensor is shown in Fig. 1(c), which clearly shows the peaks associated with the D and G bands at 1350 cm^−1^ (the dispersive, defect induced vibrations) and 1580 cm^−1^ (related to the vibration of sp2 -bonded carbon atoms)^28^.

### Receptor immobilization

**Figure 2:**
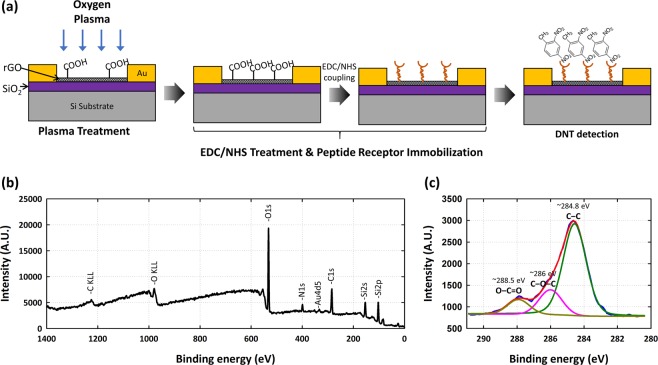
Surface functionalization of the rGO sensor: (**a**) Schematic illustration of the DNT binding peptide receptor functionalization process. (**b**) Wide-range scan XPS results of DNT-bp on the rGO sensor. (**c**) C1s region (narrow range scan) XPS results showing the resolved 284.8 eV (C-C, graphitic group), 286 eV (C-O-C, ether group) and 288.5 eV (O-C=O, carboxylic acid group) peaks, respectively.

We immobilized the peptide (aptamer) receptor by first cleaning the rGO sensor surface with ethanol, absolute phosphate-buffered saline (PBS) 1X and deionized water, sequentially. Then, we exposed the surface to O_2_ plasma using 50 W for 30 s to enhance the prevalence of carboxyl groups on the rGO surface (Fig. 2(a)). Subsequently, the carboxyl groups were subjected to EDC/NHS coupling by treatment with a mixture of 100 mM 1-ethyl-3-(3-dimethylaminopropyl)carbodiimide hydrochloridem (EDC, Sigma Aldrich, Korea)/50mM N-succinimide, (NHS, Sigma Aldrich, Korea) in PBS (2 h at RT). After incubation, we washed the rGO sensor surface using ethanol, PBS and deionized water, sequentially. Then, we immobilized the DNT-specific binding peptide (DNT-bp, sequence: His-Pro-Asn-Phe-Se r-Lys-Tyr-IleLeu-HisGln-Arg-Cys)^25^ and DNT-non-specific binding peptide (DNT-nbp, sequence: Thr-Ser-Met-Leu-Leu-Met-Ser-Pro-Lys-His-Gln-Ala-Cys) on the surface of the rGO sensor^22,23^.

X-ray photoelectron spectroscopy (XPS) was used to check whether the immobilization process was successful. An analysis of the DNT-bp on the rGO surface (In Fig. 2(b)) showed peaks at 285.3 eV for C1s and 532.6 eV for O1s, respectively. The XPS spectra of the rGO surface revealed N1s peaks (398.9 eV) across a wide range. The well-resolved spectrum of C1s, which could be deconvoluted by O-C=O binding, was analyzed to resolve the peaks with Gaussian profiling (Fig. 2(c)). This spectrum was split into peaks at 284.8 (C-C, graphitic group), 286 (C-O-C, ether group) and 288.5 eV (O-C=O, carboxylic acid group), respectively. The peaks centered at 398.9 eV were assigned to the amine nitrogen, as in a previous report, confirming the stable immobilization of antibodies on the rGO surface^29^. Moreover, the presence of the N1s peak confirms the chemical modification of the carboxylic acid group with EDC/NHS and the peptide receptor^30^.

### Measurement setup

The process of sample loading and electrical readout is schematically shown in Fig. S2 in the supplementary information. The gas generating system consisted of three mass flow controllers (MFC, TSC-D, MK Precision Inc.) and four on/off solenoid valves to control the flow (rate and direction) connected with Teflon tube lines. We exposed the target sample to a flow of 100 standard centimeter cubic per minute (sccm). We designed an array of eight rGO patterns on a chip and three types of rGO surfaces were prepared: two of them were functionalized with DNT-bp and DNT-nbp, respectively, whereas the third surface remained bare. The chip was further designed to enable electrical resistance measurements in real time by equipping the gas chamber with eight electrical probes to acquire electrical signals between the two electrodes. We fabricated the gas reaction chamber (see the supplementary information (Fig. S3)) from polyarylether-etherketone (PEEK™). We monitored the electrical resistance of the rGO pattern with a multi-channel read-out system using a KEITHLEY2410 voltage sourcemeter connected with 1 × 8 multiplex type PXI-1033 (National Instrument, USA) (Fig. S2). We used LabVIEW software (National Instrument, USA) both for controlling and measuring the electrical resistance of the rGO sensors.

We defined the changes in resistance as the DNT sensing signal using the following relation:

$$Resistance\,change\,( \% )=\frac{{R}_{after}-{R}_{before}}{{R}_{before}}\times 100$$

where R_before_ and R_after_ represent the resistance values before and after DNT exposure.

## Results and Discussion

**Figure 3:**
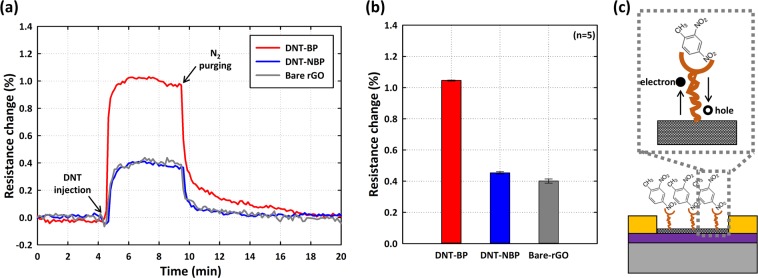
DNT detection using the DNT-bp, DNT-nbp, and bare rGO sensor surfaces: (**a**) Real-time monitoring of the resistance change with multi-functionalized rGO sensors. (**b**) Average resistance changes of five independent multi-functionalized rGO sensors. The red, blue, and gray bars represent the DNT-bp, DNT-nbp, and bare rGO sensors, respectively. The error bars indicate the standard deviation. (**c**) Schematic illustration of the equivalent effect of DNT detection with DNT-bp and rGO.

The ability of the sensor to achieve multiple sensing was confirmed by using the functionalized DNT-bp and DNT-nbp and the bare surfaces on the rGO sensor, and simultaneously monitored the resistance change (Fig. 3). The electrical signal was measured under stabilized conditions by injecting DNT gas while allowing N_2_ gas to flow for 5 min (100 sccm) to confirm that the changes in resistance with time had been stabilized without any drift. Then, we injected DNT vapor containing 320 ppb for the following 5 min, and then again sequentially injected N_2_ gas to purge the DNT vapor for 10 min. In Fig. 3(a), the resistance change of the DNT-bp functionalized rGO pattern increased sharply (~104 × 10^−4^) whereas the resistance change detected for the DNT-npb and bare functionalization surfaces was relatively small, as shown in Fig. 3(a,b). The changes in the resistance of the DNT-npb and bare functionalization were approximately 45 × 10^−4^ and 40 × 10^−4^, respectively. Interestingly, the changes in the resistance of the surface functionalized with DNT-npb and the bare surface were similar to those of DNT gas, whereas DNT-bp showed a large increase in resistance. We also observed fast recovery (~17 min) of the binding surface to restore the initial sensor surface by only using N_2_ gas.

We acquired an enhanced signal by using the differential signal between DNT-bp and DNT-nbp, because this approach enabled us to eliminate unwanted electrical noise and/or environmental effects. Therefore, differential values in the resistance change provide us with clear information of the reaction kinetics between DNT-bp and DNT gas molecules under various environmental conditions. For the resistance changes of specific interactions between DNT and DNT-bp, we speculated that the electron withdrawing from rGO to the NO_2_ group of DNT could decrease the resistance^24,31^. The NO_2_ group of DNT has been known as one of the strong electron withdrawing groups (EWG), consequently, affects the rGO surface’s resistance since it showed similar effect like the hole carrier injection (Fig. 3(c)).

**Figure 4:**
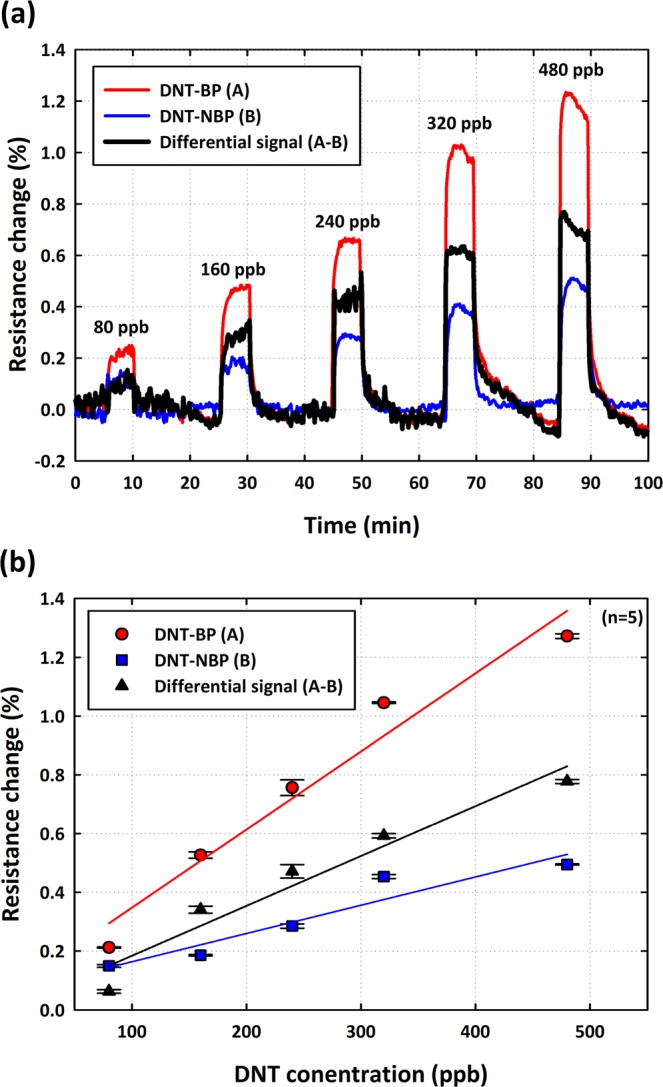
Sensitivity test: (**a**) Real-time monitoring of resistance change for different concentrations of DNT vapor. The sensing signals of both DNT-bp (red) and DNT-nbp (blue) were measured to calculate the differential signal (black). (**b**) Sensitivity results in (**a**) plotted as a function of the DNT concentration and the calculated differential, showing that the linear sensitivity of the differential signal was (0.17 ± 0.02) × 10^−4^ per ppb. The R-square values for DNT-bp, DNT-nbp and differential value are approximately 0.9590, 0.9091 and 0.9414, respectively.

We assessed the sensitivity by measuring the change in resistance of the DNT-bp and DNT-nbp functionalized rGO sensors (red and blue traces in Fig. 4(a)), and calculated the differential signal from the resistance change of DNT-bp and DNT-nbp (black trace in Fig. 4(a)). We repeated the test five times with multiple devices. We sequentially injected DNT vapor of 80, 160, 240, 320, and 480 ppm into the rGO sensor and monitored the resistance changes using the measurement system. Figure 4(a) shows that the response increased abruptly upon exposure to DNT gas. The resistance change increased proportionally to the DNT gas concentration. That is, increasing the DNT concentration from 80 to 480 ppb caused the resistance of DNT-bp to change and the following values were measured: 21 × 10^−4^, 52 × 10^−4^, 75 × 10^−4^, 104 × 10^−4^, 127 × 10^−4^ whereas the corresponding values measured for DNT-nbp were 15 × 10^−4^, 18 × 10^−4^, 28 × 10^−4^, 45 × 10^−4^, 49 × 10^−4^, respectively. In Fig. 4, we show the differential signal between DNT-bp and DNT-nbp. The differential signal (black trace in Fig. 4) was calculated by extracting the DNT-nbp signal from that of DNT-bp Consequently, we were able to obtain pure information from the specific binding of DNT gas, without the inevitable unwanted electrical noise and/or environmental effects. The differential signals were 6.2 × 10^−4^, 34 × 10^−4^, 47 × 10^−4^, 59 × 10^−4^, and 78 × 10^−4^ for 80, 160, 240, 320, and 480 ppb, respectively. We calculated the sensitivity from the resistance change versus the DNT concentration for DNT-bp, DNT-nbp, and the differential signals, showing the linear sensitivity of the differential signal calculated as (0.17 ± 0.02) × 10^−4^ per ppb (Fig. 4(b)). The differential signal provides true specific binding information and reveals a small coefficient of variance (CV) value, i.e., 1.28%, from multiple runs, confirming the reliability and reproducibility of the results.

**Figure 5:**
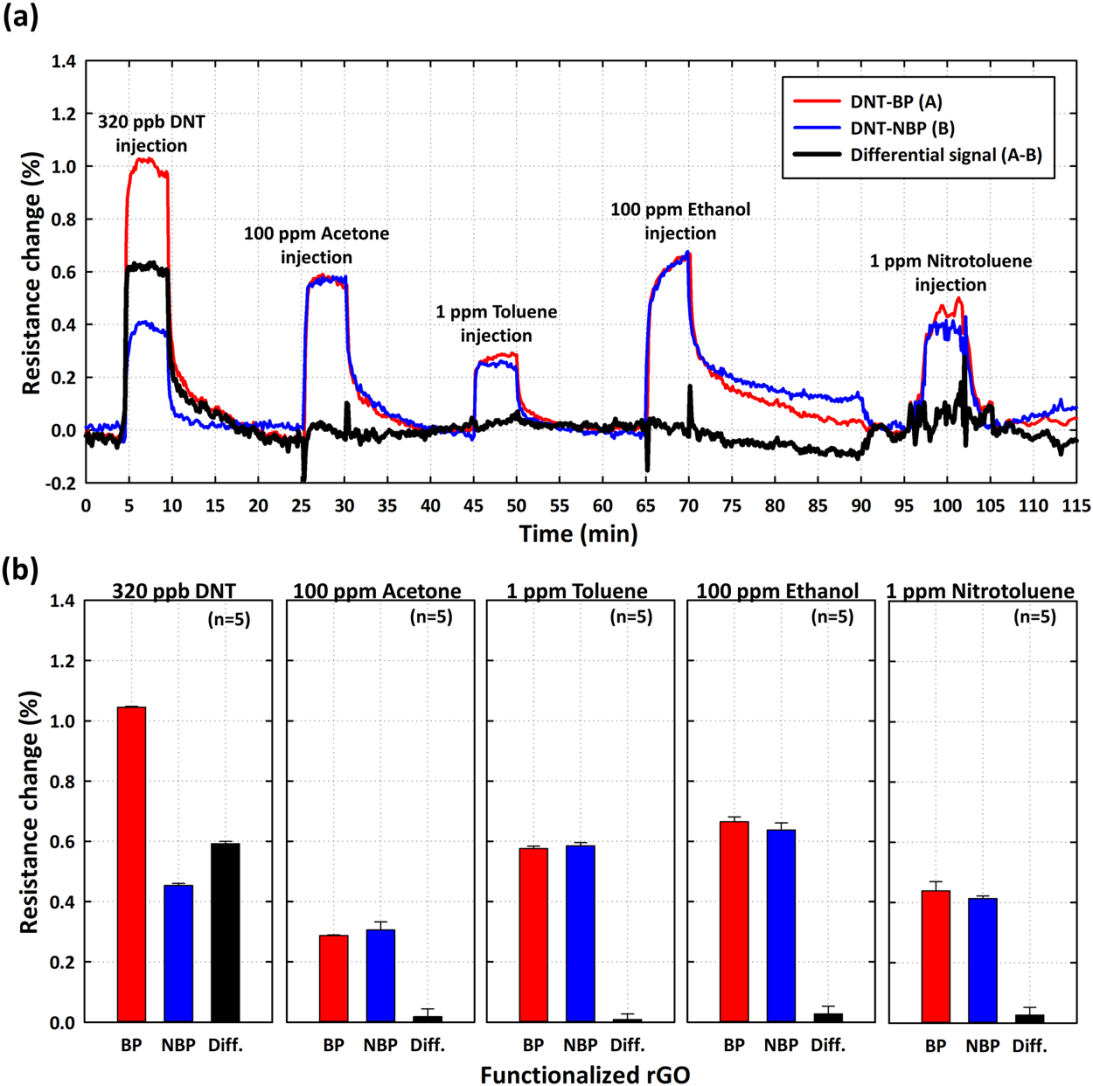
Selectivity test. (**a**) DNT-bp (red), DNT-nbp (blue), and differential signal (black) was monitored in the presence of DNT vapor of 320 ppb, acetone of 100 ppm, toluene of 1 ppm, ethanol of 100 ppm, and nitrotoluene of 1 ppm. (**b**) Average resistance change for each of the five different gases: the differential values (black bar) of DNT-bp and DNT-nbp confirm the high selectivity.

Importantly, the selectivity is an essential criterion in a chemical/bio sensor because it indicates the ability to discriminate between different substances^23^. For selectivity test, we first prepared the DNT-bp and DNT-nbp functionalized rGO gas sensors. Then we sequentially injected DNT vapor (320 ppb), acetone (100 ppm), toluene (1 ppm), ethanol (100 ppm), and nitrotoluene (1 ppm) into the rGO sensors (Fig. 5). For DNT (320 ppb), the resistance changes from the DNT-bp and DNT-nbp were 103 × 10^−4^ and 39 × 10^−4^ and the differential signal was calculated as 64 × 10^−4^. After N_2_ purging, we sequentially injected acetone (100 ppm) and observed that the differential signal is almost 0. Interestingly, the concentration of 100 ppm acetone gas exceeded that of 320 ppb DNT by 312 times, indicating that binding between DNT-bp and the peptide was highly specific. Similarly, we observed no changes in the differential signal of toluene, ethanol and nitrotoluene, further confirming the excellent selectivity for different gases (Fig. 5(b)).

**Figure 6:**
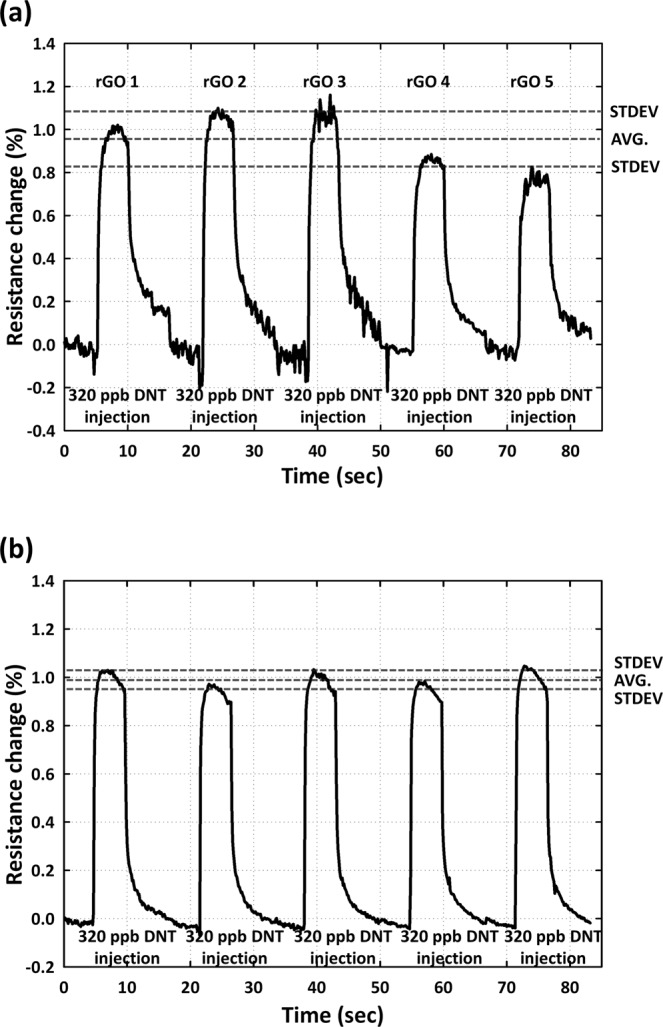
Reproducibility and sensor regeneration test for (**a**) multiple devices and (**b**) multiple runs. The resistance change measured during multiple runs is reproducible (shown here for 320 ppb DNT), and the trace in (**b**) also confirms complete regeneration of the sensor surface between successive runs at room temperature by using N_2_ gas.

The precision of the reproducibility was verified by using both multiple devices (Fig. 6(a) and multiple runs (Fig. 6(b)) to obtain measurements. Importantly, the measuring data from multiple runs could also provide an indication of the ability of the receptor surface to undergo regeneration. The requirement in terms of the regeneration step was that purging with N_2_ was only required to remove the layers of DNT gas molecules, while leaving the immobilized peptide (aptamer) undisturbed. For the test in which multiple devices were used (Fig. 6(a)), we prepared a DNT-bp rGO sensor. We first stabilized the resistance change (5 min), injected DNT vapor of 320 ppb (5 min), and then purged with N_2_ gas (10 min), sequentially. This process was repeated five times with multiple rGO sensor chips (N = 5). The averaged resistance change measured for these five rGO sensors was approximately 98.8 × 10^−4^, with a standard deviation of approximately 0.13%; furthermore, the coefficient of variation (C.V.) was calculated as 13.8% from multiple rGO sensor chips, showing good reproducibility. For multiple runs (Fig. 6(b)), we tested the reproducibility with the same rGO sensor, using the protocol suggested in Fig. 6(a). The averaged resistance change via DNT-bp and DNT gas interaction was approximately 99.6 × 10^−4^, with a standard deviation of approximately 2.3 × 10^−4^ and the coefficient of variation (C.V.) was calculated as approximately 2.30% from multiple rGO sensor chips, showing great reproducibility.

In general, the chemiresistive gas sensors based on metal oxides require external heaters to accomplish the adsorption and desorption of target gas molecules, which, in turn, increases the power consumption, hindering the practical implementation of these sensors in IoT applications^5^. We demonstrated that the peptide (aptamer) receptor for the DNT gas sensor is almost entirely regenerated at room temperature by only using N_2_ gas, showing the possibility of selective detection and the ability to regenerate sensing surfaces. The recovery times when using N_2_ gas (100 sccm) are approximately 97.4 and 284.8 s to recover the resistance change up to 70 and 100%, respectively (N = 5).

We presented a concise summary of previous studies related to graphene-based gas sensors in Table 1 to place our work in context. These previous studies pertained to the sensing material, material/fabrication technique, recovery (regeneration) method, target gas, response time/70% recovery (regeneration) time, and response/limit of detection. Therefore, compared to previous work, the proposed preconcentration platform is, firstly, capable of excellent sensitivity/selectivity up to the part per billion level. Furthermore, sensing and desorption of the target chemical at room temperature were shown to be possible; moreover, the sensor surface could be rapidly regenerated by N_2_ gas within a few minutes.

**Table 1:** Comparison of previously reported graphene-based gas sensors.

Author	Sensing material	Material/Fabrication technique	Recovery (regeneration) method	Target gas	Response time/70% Recovery (regeneration) time	Response/Limit of detection	Year/Ref.
Ganhua Lu *et al*.	Reduced graphene oxide	annealing	N_2_ purging	NO_2_	~15 min/~30 min	1.41–1565 ((G_g_-G_a_)/G_a_)/2 ppm	2009/^32^
Xiaogan Li *et al*.	Reduced graphene oxide	TiO_2_ decoration	Gas off	NH_3_	~10 min /~20 min	9.4983 Ω/10 ppm	2016/^33^
Hyeun Joong Yoon *et al*.	Graphene sheet	—	Gas off	CO_2_	~20 s/~20 sec	−/~10 ppm	2011/^34^
Min Gyun Chung *et al*.	Transferred graphene	O_3_ (ozone) treatment		NO_2_	~12–15 min/~25–30 min	9–19.7% ((R_a_-R_g_)/R_a_)/200 ppb	2012/^14^
Gaurav Singh *et al*.	Reduced graphene oxide	ZnO decoration		CO	~5 s/~2–5 s	24.3% (∆G)/22 ppm	2012/^15^
Adarsh Kaniyoor *et al*.	Graphene and multiwall carbon nanotube	Pt deconration		Hydrogen			2009/^16^
Tran Viet Cuong *et al*.	graphene	ZnO-chemically conversion		H_2_S	~1900–2000 s/~2500 s	50% ((R_g_-R_a_)/R_a_)/2 ppm	2010/^35^
Haichuan Mu *et al*.	graphene	ZnO film atomic layer deposition		formaldehyde		−/9 ppm	2014/^36^
Alexey Lipatov *et al*.	Reduced graphene oxide	Thermal reduction	Air purging	Alcohol (isopropanol, methanol, ethanol)	~5 min/~10 min		2013/^37^
R. Pearce *et al*.	Epitaxially grown graphene	graphene	Air purging	NO_2_	~1 h/~2–3 h	0.005 (R/R_0_)/2.5 ppm	2011/^13^
G. Ko *et al*.	graphene	—	Air purging	NO_2_	~70–100 s/~200–300 s	9–14% ((R_a_-R_g_)/R_a_)/100 ppm	2010/^38^
Wei Wu *et al*.	Wafer-scale synthesis graphene			H_2_	~213 s/~463 s	0.2–10% ((R_g_-R_a_)/R_a_)/0.0025%	2010/^39^
This work	Reduced graphene oxide	peptide receptor via O_2_ plasma treatment	N_2_ purging	2,4-dinitrotoluene	~10 s/~35 s	0.0027% (∆R/R_0_)/2.426 ppb	—

## Conclusion

We designed and fabricated a highly sensitive and selective DNT gas sensor using DNT-specific binding peptide functionalized rGO. We calculated the sensitivity by measuring the resistance change using the differential signals between DNT-bp and DNT-nbp. The technique showed excellent linear sensitivity of (0.27 ± 0.02) × 10^−4^ per ppb with an approximate limit of detection (LOD) of 2.43 ppb. Upon exposure to various gases such as acetone (100 ppm), toluene (1 ppm), and ethanol (100 ppm), our sensor proved to be highly selective with fast response. Moreover, we demonstrated that the surface could be completely regenerated at room temperature, showing the possibility of regenerating the binding surface without the need to consume power. For practical applications, further study is needed to reflect the real conditions that explain the effect of temperature, humidity, sample collection and shelf-life of receptor.

## Supplementary information

Supplementary Information

## References

[1] Ma Y, Li H, Peng S, Wang L (2012). Highly Selective and Sensitive Fluorescent Paper Sensor for Nitroaromatic Explosive Detection. Analytical Chemistry.

[2] Peng Gang, Tisch Ulrike, Adams Orna, Hakim Meggie, Shehada Nisrean, Broza Yoav Y., Billan Salem, Abdah-Bortnyak Roxolyana, Kuten Abraham, Haick Hossam (2009). Diagnosing lung cancer in exhaled breath using gold nanoparticles. Nature Nanotechnology.

[3] Schedin F., Geim A. K., Morozov S. V., Hill E. W., Blake P., Katsnelson M. I., Novoselov K. S. (2007). Detection of individual gas molecules adsorbed on graphene. Nature Materials.

[4] Zhang Y (2018). Sensing methamphetamine with chemiresistive sensors based on polythiophene-blended single-walled carbon nanotubes. Sensors and Actuators B: Chemical.

[5] Kwak S (2018). MEMS-Based Gas Sensor Using PdO-Decorated TiO2 Thin Film for Highly Sensitive and Selective H2 Detection with Low Power Consumption. Electronic Materials Letters.

[6] Dey A (2018). Semiconductor metal oxide gas sensors: A review. Materials Science and Engineering: B.

[7] Xia Yi, Li Ran, Chen Ruosong, Wang Jing, Xiang Lan (2018). 3D Architectured Graphene/Metal Oxide Hybrids for Gas Sensors: A Review. Sensors.

[8] Nasir Salisu, Hussein Mohd, Zainal Zulkarnain, Yusof Nor (2018). Carbon-Based Nanomaterials/Allotropes: A Glimpse of Their Synthesis, Properties and Some Applications. Materials.

[9] Sun Y-P, Fu K, Lin Y, Huang W (2002). Functionalized Carbon Nanotubes:  Properties and Applications. Accounts of Chemical Research.

[10] Kim Keun Soo, Zhao Yue, Jang Houk, Lee Sang Yoon, Kim Jong Min, Kim Kwang S., Ahn Jong-Hyun, Kim Philip, Choi Jae-Young, Hong Byung Hee (2009). Large-scale pattern growth of graphene films for stretchable transparent electrodes. Nature.

[11] Deng S (2012). Reduced Graphene Oxide Conjugated Cu2O Nanowire Mesocrystals for High-Performance NO_2_ Gas Sensor. Journal of the American Chemical Society.

[12] Dua V (2010). All-Organic Vapor Sensor Using Inkjet-Printed Reduced Graphene Oxide. Angewandte Chemie.

[13] Pearce R (2011). Epitaxially grown graphene based gas sensors for ultra sensitive NO_2_ detection. Sensors and Actuators B: Chemical.

[14] Chung MG (2012). Highly sensitive NO_2_ gas sensor based on ozone treated graphene. Sensors and Actuators B: Chemical.

[15] Singh G (2012). ZnO decorated luminescent graphene as a potential gas sensor at room temperature. Carbon.

[16] Kaniyoor A, Imran Jafri R, Arockiadoss T, Ramaprabhu S (2009). Nanostructured Pt decorated graphene and multi walled carbon nanotube based room temperature hydrogen gas sensor. Nanoscale.

[17] Esfandiar A, Ghasemi S, Irajizad A, Akhavan O, Gholami MR (2012). The decoration of TiO2/reduced graphene oxide by Pd and Pt nanoparticles for hydrogen gas sensing. International Journal of Hydrogen Energy.

[18] Oh YJ (2014). Oxygen functional groups and electrochemical capacitive behavior of incompletely reduced graphene oxides as a thin-film electrode of supercapacitor. Electrochimica Acta.

[19] Chae M-S (2017). Enhancing surface functionality of reduced graphene oxide biosensors by oxygen plasma treatment for Alzheimer’s disease diagnosis. Biosensors and Bioelectronics.

[20] Masaki H, Yuki H, Yasuhide O, Kenzo M, Kazuhiko M (2014). Characterization of reduced graphene oxide field-effect transistor and its application to biosensor. Japanese Journal of Applied Physics.

[21] Cai B (2014). Ultrasensitive Label-Free Detection of PNA–DNA Hybridization by Reduced Graphene Oxide Field-Effect Transistor Biosensor. ACS Nano.

[22] Hwang KS (2011). Peptide receptor-based selective dinitrotoluene detection using a microcantilever sensor. Biosensors and Bioelectronics.

[23] Yoo YK (2012). Multifunctionalized Cantilever Systems for Electronic Nose Applications. Analytical Chemistry.

[24] Cui Y (2010). Chemical Functionalization of Graphene Enabled by Phage Displayed Peptides. Nano Letters.

[25] Jaworski JW, Raorane D, Huh JH, Majumdar A, Lee S-W (2008). Evolutionary Screening of Biomimetic Coatings for Selective Detection of Explosives. Langmuir.

[26] Hui Kim S (2012). Effects of water molecules on binding kinetics of peptide receptor on a piezoelectric microcantilever. Applied Physics Letters.

[27] Lee JH, Hwang KS, Kim TS (2010). The Microscopic Origin of Residual Stress for Flat Self-Actuating Piezoelectric Cantilevers. Nanoscale Res Lett.

[28] Chandra V, Kim KS (2011). Highly selective adsorption of Hg2+ by a polypyrrole–reduced graphene oxide composite. Chemical Communications.

[29] Yue J, Epstein AJ (1991). XPS study of self-doped conducting polyaniline and parent systems. Macromolecules.

[30] An Y, Chen M, Xue Q, Liu W (2007). Preparation and self-assembly of carboxylic acid-functionalized silica. Journal of Colloid and Interface Science.

[31] Yuan W, Liu A, Huang L, Li C, Shi G (2013). High-Performance NO_2_ Sensors Based on Chemically Modified Graphene. Advanced Materials.

[32] Ganhua L, Leonidas EO, Junhong C (2009). Reduced graphene oxide for room-temperature gas sensors. Nanotechnology.

[33] Li X (2016). Reduced graphene oxide (rGO) decorated TiO2 microspheres for selective room-temperature gas sensors. Sensors and Actuators B: Chemical.

[34] Yoon HJ (2011). Carbon dioxide gas sensor using a graphene sheet. Sensors and Actuators B: Chemical.

[35] Cuong TV (2010). Solution-processed ZnO-chemically converted graphene gas sensor. Materials Letters.

[36] Mu H (2014). High sensitive formaldehyde graphene gas sensor modified by atomic layer deposition zinc oxide films. Applied Physics Letters.

[37] Lipatov A (2013). Highly selective gas sensor arrays based on thermally reduced graphene oxide. Nanoscale.

[38] Ko G (2010). Graphene-based nitrogen dioxide gas sensors. Current Applied Physics.

[39] Wu W (2010). Wafer-scale synthesis of graphene by chemical vapor deposition and its application in hydrogen sensing. Sensors and Actuators B: Chemical.

