# Supplementary material for: Highly selective reduced graphene oxide (rGO) sensor based on a peptide aptamer receptor for detecting explosives
Source: Sci Rep. 2019 Jul 16;9:10297. doi: 10.1038/s41598-019-45936-z (PMC6635493; doi:10.1038/s41598-019-45936-z)
Supplement: Supplementary file 1 — Supplementary Information [file 41598_2019_45936_MOESM1_ESM.docx]

**Highly selective reduced graphene oxide (rGO) sensor based on a peptide aptamer receptor for detecting explosives**

Kyungjae Lee ^a*^, Yong Kyoung Yoo ^a*^, Myung-Sic Chae ^b^, Kyo Seon Hwang ^b^,

Junwoo Lee ^a^, Hyungsuk Kim ^a^, Don Hur ^a^ and Jeong Hoon Lee^a^

^a^Department of Electrical Engineering Kwangwoon University, 447-1 Wolgye, Nowon, Seoul 01897, South Korea

^b^Department of Clinical Pharmacology and Therapeutics, College of Medicine, Kyung Hee University, Seoul 02447, South Korea

^*^ These authors contributed equally to this work.

Correspondence and requests for materials should be addressed to J.H.L. (email: [jhlee@kw.ac.kr](mailto:jhlee@kw.ac.kr)) or D.H. (email: [dhur@kw.ac.kr](mailto:dhur@kw.ac.kr)).

**Device fabrication**


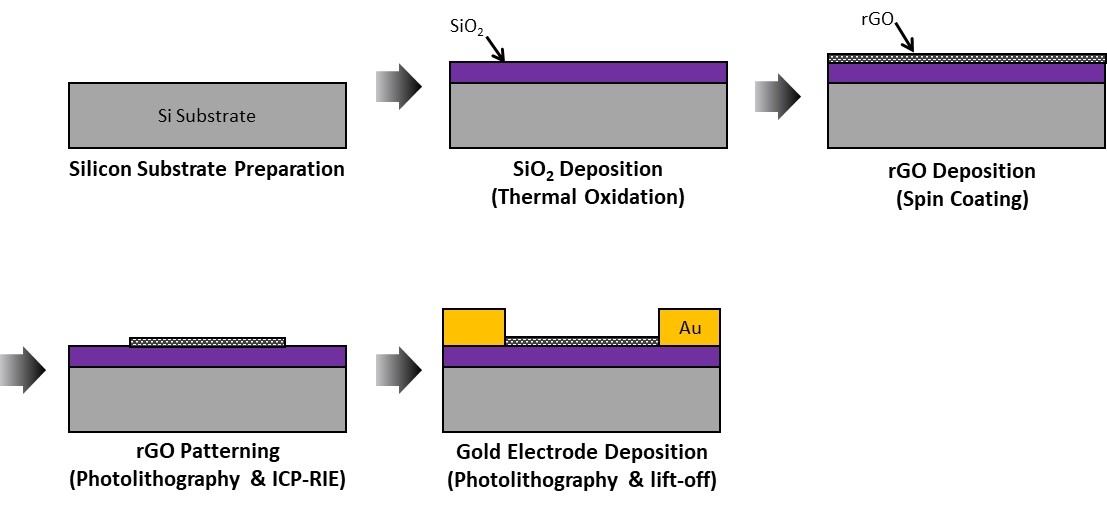


**Figure S1.** Reduced graphene oxide based gas sensor fabrication.

First, the silicon dioxide (SiO_2_) of 300 nm on the silicon wafer was deposited by thermal oxidation. Graphene oxide (GO) solution was deposited by spin coating. GO layer was exposure by hydriodic acid (HI) vapor for the reduction at 80 °C for 3 hours. After reduction, the rGO layer was patterned by photolithography and inductively coupled-plasma reactive ion etcher (ICP-RIE). Lastly, Au electrodes was formed by lift-off process.

**Gas generating and electrical measuring system**


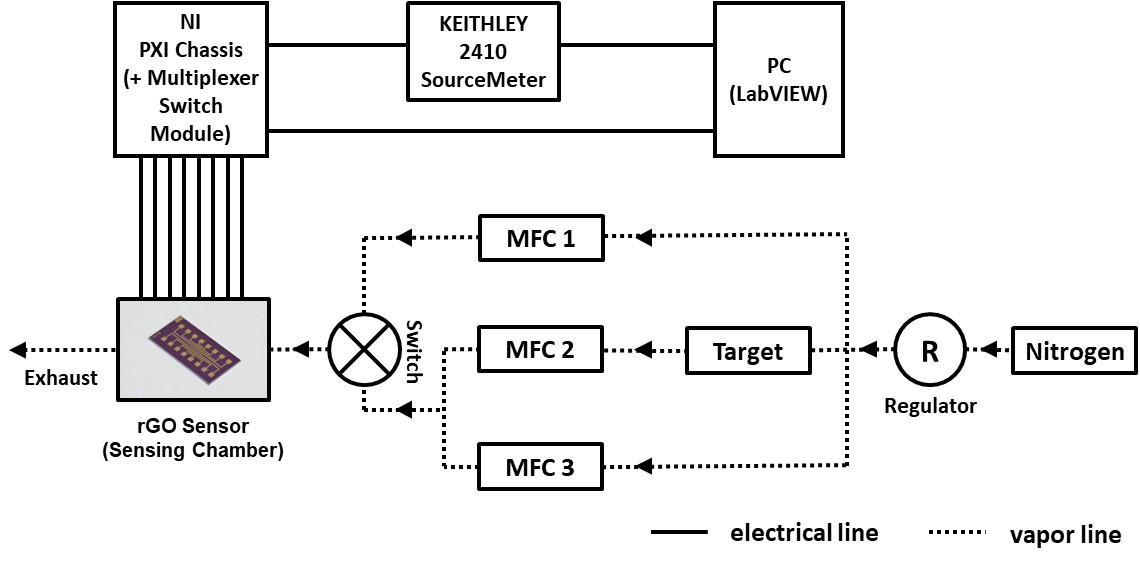


**Figure S2.** Gas generating system and electrical measuring system.

The gas generating system is consist of 3 mass flow controller (MFC), solenoid valve, regulator and target source. Each components are connected with Teflon gas tube (the dashed line represents vapor line). The target gas is delivered to rGO sensor by nitrogen carrier. And The electrical measuring system is consist of Keithley benchtop voltage source meter (KEITHLEY 2410), National Instrument multiplexer, and LabVIEW. Each components and electrical signal are controlled and acquired by LabVIEW. The solid line indicates electrical line.

**rGO sensing chamber**


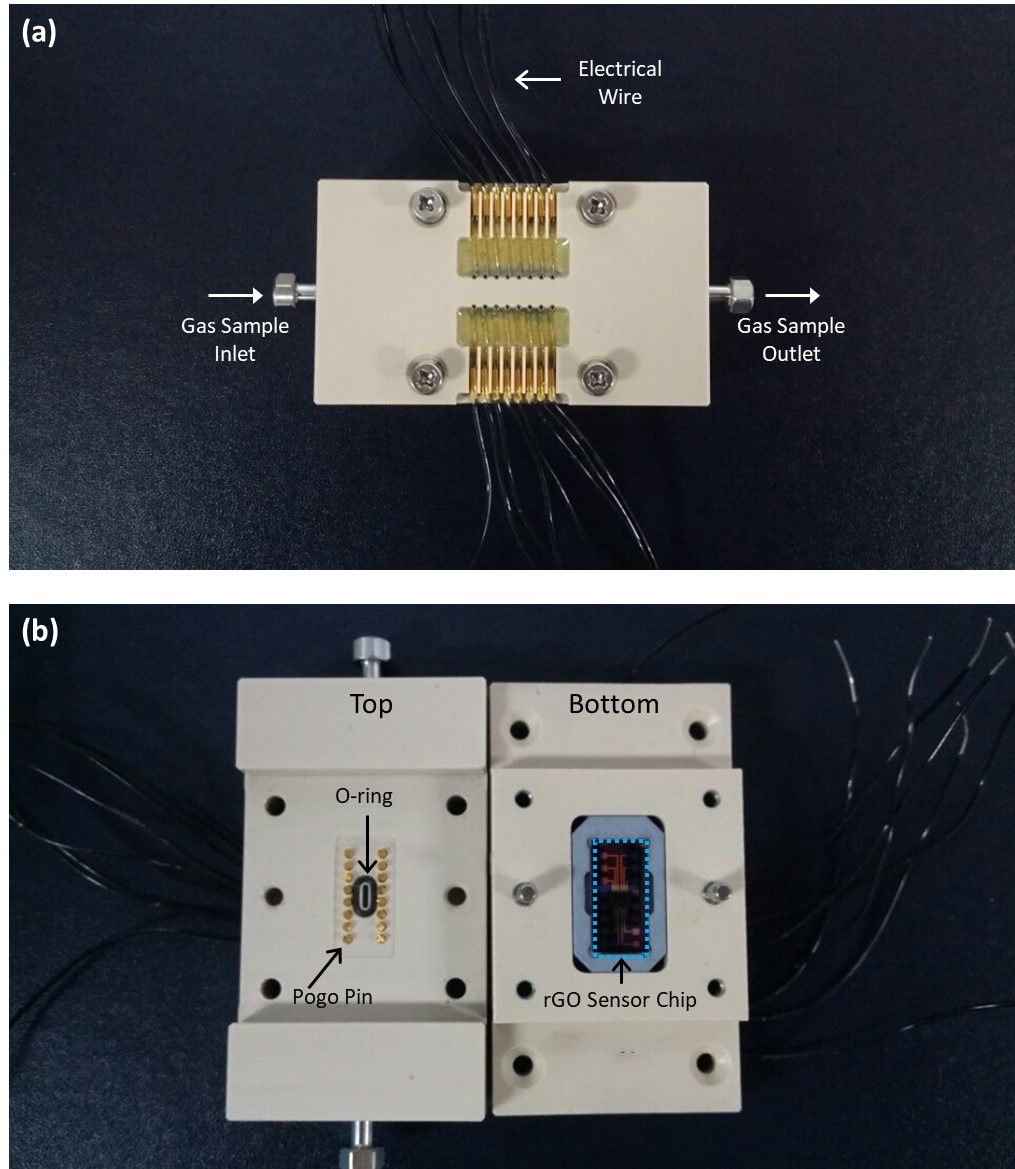


**Figure S3.** The image of rGO sensing chamber. (a) The assembled rGO sensing chamber. (b) The disassembled rGO sensing chamber. rGO sensor chip is loaded on the bottom.

**The linear response of sensitivity test according to DNT concentration.**

**Table S**1. The estimation of linear response (Fig. 4) of sensitivity test according to DNT concentration.

|  | **Equation** | **R-square** |
| --- | --- | --- |
| **DNT-bp (A)** | $y=\left( 0.0027\pm0.0003 \right)x+(0.0817\pm0.0923)$ | 0.9590 |
| **DNT-nbp (B)** | $y=\left( 0.0010\pm0.0002 \right)x+(0.0674\pm0.0510)$ | 0.9091 |
| **Differential (A-B)** | $y=\left( 0.0017\pm0.0002 \right)x+(0.0142\pm0.0711)$ | 0.9414 |
